# Supplementary material for: Phase-field modeling and machine learning of electric-thermal-mechanical breakdown of polymer-based dielectrics
Source: Nat Commun. 2019 Apr 23;10:1843. doi: 10.1038/s41467-019-09874-8 (PMC6478924; doi:10.1038/s41467-019-09874-8)
Supplement: Supplementary file 1 — Supplementary Information [file 41467_2019_9874_MOESM1_ESM.pdf]

Supplementary Information

Phase-Field Modeling and Machine Learning of Electric-Thermal-Mechanical Breakdown of Polymer-based Dielectrics

Shen et al.

Supplementary Methods

Phase-Field Model of Electrical-Thermal-Mechanical Breakdown

In the phase-field model, a continuous phase-field variable $\eta(\mathbf{r},t)$ is introduced to describe the spatial and temporal evolution of the breakdown phase: $\eta(\mathbf{r},t)$=1 represents the breakdown phase, $\eta(\mathbf{r},t)$=0 represents the non-breakdown phase, and the diffuse transitional region represents the interface area, respectively. Considering that the breakdown may arise from the electric stimuli, the mechanical stimuli, and the thermal stimuli, herein we combine the electric, thermal, and mechanical stimuli together to investigate their effects on the breakdown process. Three materials features including the dielectric constant, electrical conductivity and Young's modulus, are parameterized in this model to calculate different energies. The dielectric constant inhomogeneity of the nanocomposite is defined according to the different dielectric constants of the polymer phase, filler phase, and the breakdown phase. This also applies to the electrical conductivity and Young's modulus inhomogeneities.

The free energy considering synergistic contributions from the phase separation, the interface, the temperature and the electric field in a dielectric inhomogeneous system is written as

$$F = \int_V \left[f_{\text{sep}}\left(\eta(\mathbf{r})\right) + f_{\text{grad}}\left(\eta(\mathbf{r})\right) + f_{\text{elec}}(\mathbf{r}) + f_{\text{Joule}}(\mathbf{r}) + f_{\text{strain}}(\mathbf{r}) \right] dV, \quad (1)$$

where the first term in the integral represents the free energy density of mixing that drives the phase separation, the second term is the gradient energy density with γ the gradient energy coefficient in isotropic approximation, the third term is the electric energy density, the fourth term is the Joule heat energy density, and the last term is the strain energy density.

The gradient energy density f_{grad} is described by

$$f_{\text{grad}} = -\frac{1}{2}\gamma \left| \nabla \eta(\mathbf{r}) \right|^2, \quad (2)$$

A double-well function is used to describe the phase separation energy,

$$f_{\text{sep}}\left(\eta(\mathbf{r})\right)=\alpha\eta^2\left(1-\eta\right)^2, \tag{3}$$

with α a positive coefficient defining the energy barrier of the phase separation.

The electric energy density f_{elec} can be written as

$$f_{\text{elec}}=\frac{1}{2}\varepsilon_0\varepsilon_{ij}\left(\mathbf{r}\right)E_i\left(\mathbf{r}\right)E_j\left(\mathbf{r}\right)+\frac{1}{2}E_i\left(\mathbf{r}\right)P_i^{\text{S}}\left(\mathbf{r}\right)\ , \tag{4}$$

where $\varepsilon_{\text{ij}}\left(\mathbf{r}\right)$ is the spatially dependent relative dielectric constant tensor, $E_i\left(\mathbf{r}\right)$ is the total electric field component, and $P_i^{\text{S}}\left(\mathbf{r}\right)$ is the spontaneous polarization which is not zero if the local material component is ferroelectric.

The Joule heat energy density f_{Joule} is expressed by

$$f_{\text{Joule}}=\sigma_{ij}\left(\mathbf{r},T\right)E_i\left(\mathbf{r}\right)E_j\left(\mathbf{r}\right)dt \tag{5}$$

where $\sigma_{ij}\left(\mathbf{r},T\right)$ is the spatially and temperature dependent electrical conductivity tensor, and *dt* is the operating time of applied electric field.

According to the filamentary electromechanical breakdown mechanism, the effect of the electric field in inducing mechanical stress is also considered in this model. Here, the mechanical compressive stress induced by an electric field is $\sigma_{\text{m}}=\frac{1}{2}\varepsilon_0\varepsilon_r E^2$ [1]. So, the strain energy density f_{stain} can be written as

$$f_{\text{strain}}=\frac{\sigma_{\text{m}}^2}{2Y\left(\mathbf{r}\right)}=\frac{\varepsilon_0^2\varepsilon_{\text{ij}}^2\left(\mathbf{r}\right)E_i^2\left(\mathbf{r}\right)E_j^2\left(\mathbf{r}\right)}{8Y\left(\mathbf{r}\right)}\ , \tag{6}$$

where $Y\left(\mathbf{r}\right)$ represents the Young's modulus.

A modified Allen-Cahn equation is employed to describe the breakdown phase evolution,

$$\frac{\partial\eta\left(\mathbf{r},t\right)}{\partial t}=-L_0H\left(f_{\text{elec}}+f_{\text{Joule}}+f_{\text{strain}}-f_{\text{critical}}\right)\begin{bmatrix}\frac{\partial f_{\text{sep}}\left(\eta\right)}{\partial\eta\left(\mathbf{r},t\right)}+\frac{\partial f_{\text{grad}}\left(\mathbf{r}\right)}{\partial\eta\left(\mathbf{r},t\right)}+ \\ \frac{\partial f_{\text{elec}}\left(\mathbf{r}\right)}{\partial\eta\left(\mathbf{r},t\right)}+\frac{\partial f_{\text{Joule}}\left(\mathbf{r}\right)}{\partial\eta\left(\mathbf{r},t\right)}+\frac{\partial f_{\text{strain}}\left(\mathbf{r}\right)}{\partial\eta\left(\mathbf{r},t\right)}\end{bmatrix}, \tag{7}$$

where L_0 is the kinetic coefficient relating to the interface mobility with a value of 1.0 $m^2\ s^{-1}\ N^{-1}$, $H(f_{elec}+f_{Joule}+f_{strain}-f_{critical})$ is the Heaviside unit step function ($H((f_{elec}+f_{Joule}+f_{strain})<f_{critical})=0$ and $H((f_{elec}+f_{Joule}+f_{strain})>f_{critical})=1$), and $f_{critical}$ is a position-dependent material constant relating to the maximal energy density of each component in the nanocomposite. The purpose of introducing the Heaviside function into the Allen-Cahn equation is to assure that the breakdown phase can grow only if the electrostatic and Joule heat and strain energy of a local point is greater than its maximal energy endurance. In order to solve Eq. (7) more efficiently in Fourier space, it can be rewritten as following:

$$\frac{\eta(\mathbf{r},t+\Delta t)-\eta(\mathbf{r},t)}{\Delta t}=-L_0H\left(f_{\text{elec}}+f_{\text{Joule}}+f_{\text{strain}}-f_{\text{critical}}\right)\begin{bmatrix}\dfrac{\partial f_{\text{sep}}(\eta)}{\partial\eta(\mathbf{r},t)}+\dfrac{\partial f_{\text{elec}}}{\partial\eta(\mathbf{r},t)}\\+\dfrac{\partial f_{\text{Joule}}}{\partial\eta(\mathbf{r},t)}+\dfrac{\partial f_{\text{strain}}}{\partial\eta(\mathbf{r},t)}\end{bmatrix}$$
$$+L_0\left(H\left(f_{\text{elec}}+f_{\text{Joule}}+f_{\text{strain}}-f_{\text{critical}}\right)-\frac{1}{2}\right)\gamma\nabla^2\eta(\mathbf{r},t)+\frac{1}{2}L_0\gamma\nabla^2\eta(\mathbf{r},t+\Delta t). \tag{8}$$

Performing Fourier transformation on both sides of Eq. (8) and making rearrangement, we have

$$\tilde{\eta}(\mathbf{q},t+\Delta t)=\tilde{\eta}(\mathbf{q},t)+\frac{L_0\Delta t}{\left(1+0.5L_0\gamma\Delta tq^2\right)}\Bigg\{-H\left(f_{\text{elec}}+f_{\text{Joule}}+f_{\text{strain}}-f_{\text{critical}}\right)\times$$
$$\left[\frac{\partial f_{\text{sep}}(\eta)}{\partial\eta(\mathbf{r},t)}+\left\{\gamma q^2\tilde{\eta}(\mathbf{q},t)\right\}_{\mathbf{r}}+\frac{\partial f_{\text{elec}}}{\partial\eta(\mathbf{r},t)}+\frac{\partial f_{\text{Joule}}}{\partial\eta(\mathbf{r},t)}+\frac{\partial f_{\text{strain}}}{\partial\eta(\mathbf{r},t)}\right]_{\mathbf{q}}\Bigg\}, \tag{9}$$

where $\{\ \}_{\mathbf{q}}$ and $\{\ \}_{\mathbf{r}}$ indicate the forward and inverse Fourier transforms, respectively, and $q^2=\mathbf{q}\cdot\mathbf{q}$ is the module square of the wave vector in Fourier space.

The driving force of the order parameter evolution from the phase separation term in Eq. (7) is given by

$$\frac{\partial f_{\text{sep}}(\eta)}{\partial\eta(\mathbf{r},t)}=2\alpha\eta(1-\eta)(1-2\eta). \tag{10}$$

In order to describe the matrix and the fillers in the nanocomposite, a non-evolving field variable $\rho(\mathbf{r})$ is introduced, which is equal to 1 in the fillers and equal to 0 in the matrix. With this definition, the spatially

dependent relative dielectric constant during the microstructure evolution process takes the form:

$$\begin{aligned}\varepsilon_{ij}(\mathbf{r})=&\eta^3\left(10-15\eta+6\eta^2\right)\varepsilon_{ij}^{\mathrm{B}}+\left[1-\eta^3\left(10-15\eta+6\eta^2\right)\right]\times\\&\left\{\rho^3\left(10-15\rho+6\rho^2\right)\varepsilon_{ij}^{\mathrm{F}}+\left[1-\rho^3\left(10-15\rho+6\rho^2\right)\right]\varepsilon_{ij}^{\mathrm{M}}\right\}\end{aligned}\quad, \tag{11}$$

where $\varepsilon_{ij}^{\mathrm{B}}$, $\varepsilon_{ij}^{\mathrm{F}}$, and $\varepsilon_{ij}^{\mathrm{M}}$ indicate the relative dielectric constant of the breakdown phase, the filler, and the matrix, respectively. With the definition of the relative dielectric constant in Eq. (11), the driving force of the order parameter evolution from the electric term in Eq. (7) can be calculated as follows

$$\begin{aligned}\frac{\partial f_{\mathrm{elec}}}{\partial\eta(\mathbf{r},t)}=&15\eta^2(\eta-1)^2\varepsilon_0E_i(\mathbf{r})E_j(\mathbf{r})\times\\&\left(\varepsilon_{ij}^{\mathrm{B}}-\left\{\rho^3\left(10-15\rho+6\rho^2\right)\varepsilon_{ij}^{\mathrm{F}}+\left[1-\rho^3\left(10-15\rho+6\rho^2\right)\right]\varepsilon_{ij}^{\mathrm{M}}\right\}\right)\end{aligned}\quad. \tag{12}$$

Similarly, the spatially dependent electrical conductivity and the driving force of the order parameter evolution from the Joule heat in Eq. (7) can be expressed respectively by

$$\begin{aligned}\sigma_{ij}(\mathbf{r})=&\eta^3\left(10-15\eta+6\eta^2\right)\sigma_{ij}^{\mathrm{B}}+\left[1-\eta^3\left(10-15\eta+6\eta^2\right)\right]\times\\&\left\{\rho^3\left(10-15\rho+6\rho^2\right)\sigma_{ij}^{\mathrm{F}}+\left[1-\rho^3\left(10-15\rho+6\rho^2\right)\right]\sigma_{ij}^{\mathrm{M}}\right\}\end{aligned}, \tag{13}$$

$$\begin{aligned}\frac{\partial f_{\mathrm{Joule}}}{\partial\eta(\mathbf{r},t)}=&30\eta^2(\eta-1)^2E_i(\mathbf{r})E_j(\mathbf{r})dt\times\\&\left(\sigma_{ij}^{\mathrm{B}}-\left\{\rho^3\left(10-15\rho+6\rho^2\right)\sigma_{ij}^{\mathrm{F}}+\left[1-\rho^3\left(10-15\rho+6\rho^2\right)\right]\sigma_{ij}^{\mathrm{M}}\right\}\right)\end{aligned}, \tag{14}$$

where the σ_{ij}^{B}, σ_{ij}^{F}, and σ_{ij}^{M} indicate the electrical conductivity of the breakdown phase, the filler, and the matrix, respectively.

In a similar way, the Young's modulus and driving force of strain energy in Eq. (7) can be written respectively by,

$$\begin{aligned}Y(\mathbf{r})=&\eta^3\left(10-15\eta+6\eta^2\right)Y^{\mathrm{B}}+\left[1-\eta^3\left(10-15\eta+6\eta^2\right)\right]\times\\&\left\{\rho^3\left(10-15\rho+6\rho^2\right)Y^{\mathrm{F}}+\left[1-\rho^3\left(10-15\rho+6\rho^2\right)\right]Y^{\mathrm{M}}\right\}\end{aligned}, \tag{15}$$

$$\frac{\partial f_{\text{strain}}}{\partial \eta(\mathbf{r},t)}=2\varepsilon^2{}_0\varepsilon_{ij}(\mathbf{r})E_i^2(\mathbf{r})E_j^2(\mathbf{r})/(8Y(\mathbf{r}))*\frac{\partial \varepsilon_{ij}(\mathbf{r})}{\partial \eta(\mathbf{r},t)}$$
$$-\frac{1}{8Y^2(\mathbf{r})}\varepsilon^2{}_0\varepsilon^2{}_{ij}(\mathbf{r})E_i^2(\mathbf{r})E_j^2(\mathbf{r})*\frac{\partial Y(\mathbf{r})}{\partial \eta(\mathbf{r},t)}, \quad (16)$$

where the Y^{B}, Y^{F}, and Y^{M} indicate the Young's modulus of the breakdown phase, the filler, and the matrix, respectively.

In order to obtain the electric field distribution during the microstructure evolution process, the spectral iterative perturbation method is employed with the iterative equation in real space as following,

$$\sigma_{ij}^{\text{ref}}\frac{\partial^2\varphi^n(\mathbf{r})}{\partial r_i\partial r_j}=\frac{\partial}{\partial r_i}\left[\Delta\sigma_{ij}(\mathbf{r})E_j^{\text{ext}}\right] \quad (17)$$

with σ_{ij}^{ref} a homogeneous reference of the electrical conductivity, and $\Delta\sigma_{ij}(\mathbf{r})=\sigma_{ij}(\mathbf{r})-\sigma_{ij}^0$ the inhomogeneous perturbation. With Eq. (9) being evolved by a single step, Eq. (17) is iterated to a quasi-stationary solution with a relative error between two steps smaller than 10^{-4}.

In the phase-field simulation, the characteristic length scale $d_0=\sqrt{\gamma/\alpha}$ and the characteristic time scale $t_0=1/(L_0\alpha)$ can be defined in terms of the material parameters γ, α and L_0. The L_0 is assigned a value of 1.0 $\mathrm{m^2\ s^{-1}\ N^{-1}}$ due to the lack of experimental data. A grid size of $N_x\Delta x\times N_y\Delta x\times 1$with grid space of $\Delta x=d_0$ is employed in all simulations. For all 2-dimensional simulations, $N_x=N_y=256$, $N_z=1$. A time interval $\Delta t=0.01t_0$ is used to numerically evolve Eq. (8). As an example, the parameters of pure P(VDF-HFP) polymer: dielectric constant, electrical conductivity, Young's modulus and breakdown strength, are obtained from experiments[2,3], as shown in Supplementary Table 1. The relative dielectric constant ε^{B} and the electrical conductivity σ^{B} of the breakdown phase are considered to be isotropic and to have a value of 10^3 and 1×10^{-5} S $\mathrm{m^{-1}}$ to reflect its abundant space charges in the breakdown region, while Young's modulus of the breakdown phase is regarded stable in this model. The critical energy of each component in the nanocomposite is calculated by $f_{\text{critical}}=\frac{1}{2}\varepsilon_0\varepsilon_{\mathrm{r}}E_{\mathrm{b}}^2+\sigma E_{\mathrm{b}}^2dt+\frac{\varepsilon_0^2\varepsilon_{\mathrm{r}}^2E_{\mathrm{b}}^4}{8Y}$. The breakdown strength of

P(VDF-HFP) polymer is temperature dependent due to low tolerance to high temperature of polymer, as shown in Supplementary Table 1. Due to the lack of experimental data on the intrinsic breakdown strength of P(VDF-HFP), a thermochemical description of the ultimate breakdown strength $E_b = 35\varepsilon^{-0.64} \left(\mathrm{MV\ m^{-1}}\right)$ is used to calculated the theoretical breakdown value[4]. The coefficient α in the separation energy term is given a value of 10^8 J m^{-3} to represent the barrier of the breakdown phase and the non-breakdown phase. A value of 10^{-10} J m^{-1} is used for the gradient energy coefficient γ to specify d_0 1 nm in the modeling[5].

Supplementary Table 1 The parameters of P(VDF-HFP) used in the phase-field simulation

Temperature (K)	Dielectric constant	Electrical conductivity (S m^{-1})	Young's modulus (MPa)	Intrinsic breakdown strength (kV mm^{-1})
295	13.5	2.12e-10	982	692
313	15	/	669	619
323	16	/	537	580
333	16.3	5.1e-9	426	494
343	15.9	/	336	507
353	15.4	/	275	450
363	15.2	5e-8	214	429

Regression Analysis

A detailed workflow of regression analysis of the breakdown strength is shown in Supplementary Figure 1. Here, we fix the matrix of P(VDF-HFP) polymer and take the material parameters of filler as variables. As the first step, based on the phase-field model, 343 breakdown strength values of different nanocomposites are calculated as the training set. Three features including the dielectric constant, electrical conductivity and Young's modulus are chosen as the fingerprints to represent different fillers, and 12 prototypical functions are combined with one, two or three fingerprints to form new compound descriptors. For example, a type of the compound descriptors with three fingerprints can be permutation and combination of any three prototypical functions. As shown in Supplementary Figure 1, 1st screening is performed by ranking the coefficient of determination R^2 by Least Squares Regression (LSR) to find the best combination of each fingerprint with one function.

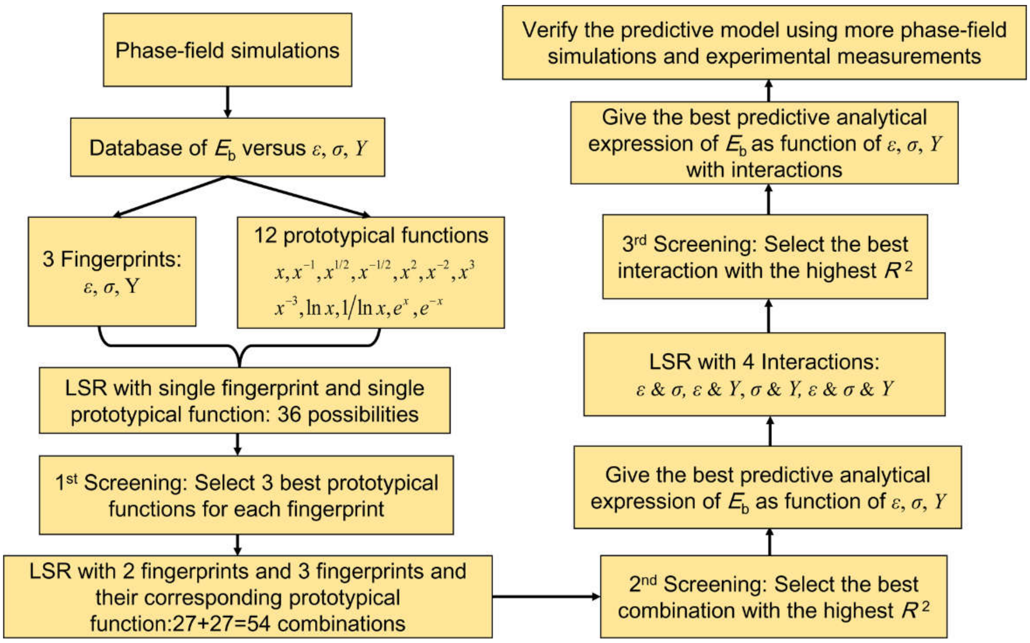

Supplementary Figure 1 Detailed workflow of regression analysis.

Supplementary Table 2 shows the regression results of combinations of each fingerprint with one function in 1st screening. It is found that all regression results of one fingerprint combined with only one

prototypical function exhibit low R^2, of which ln(x) function can lead to better R^2=0.02306 and R^2=0.6225 for dielectric constant ε and electrical conductivity σ than other functions. The Young's modulus Y shows a best R^2=0.2443 with $x^{-1/2}$ function. What's more, the breakdown strength is more dependent on electrical conductivity and Young's modulus. Based on the 1st screening, top three functions for each fingerprint are selected to compose the compound descriptors with two and three dependent fingerprints in 2nd round regression and screening. The regression results are shown in Supplementary Tables 3 and 4. For two fingerprints, electrical conductivity σ with ln(x) function and Young's Modulus Y with $x^{-1/2}$ function can result in a better R^2=0.8668. Furthermore, three fingerprints with three best prototypical functions are performed to find the best predictive expression. As shown in Supplementary Table 4, ε & σ &T with orderly ln(x) & ln(x) & $x^{-1/2}$ functions exhibits the best R^2=0.8899.

Supplementary Table 2. The coefficient of determination R^2 in 1st round of least square regressions with only one fingerprint and one of the 12 prototypical functions.

Descriptor	R^2 (only ε)	R^2 (only σ)	R^2 (only Y)
x	0.0146	0.4111	0.04036
x^{-1}	0.01204	0.2139	0.1968
$x^{1/2}$	0.01788	0.5176	0.06830
$x^{-1/2}$	0.01523	0.3059	0.2443
x^2	0.01323	0.3670	0.03278
x^{-2}	0.01082	0.1834	0.1755
x^3	0.01310	0.3627	0.03212
x^{-3}	0.01070	0.1805	0.1734
ln(x)	0.02306	0.6225	0.2312
ln(x)$^{-1}$	/	/	/
e^x	/	/	/
e^{-x}	0.01681	0.4924	0.2176

Supplementary Table 3. The coefficient of determination R^2 in 2nd round of least square regressions with two fingerprint and corresponding three best prototypical functions selected from Supplementary Table 2.

Descriptor	R^2 (ε & σ)	Descriptor	R^2(σ &Y)	Descriptor	R^2(ε&Y)
ln(x)&ln(x)	0.6456	ln(x)&ln(x)	0.8537	ln(x)&ln(x)	0.2543
ln(x)&e^{-x}	0.5155	ln(x)&e^{-x}	0.8401	ln(x)&e^{-x}	0.2407
ln(x)&$x^{1/2}$	0.5407	ln(x)&$x^{-1/2}$	0.8668	ln(x)&$x^{-1/2}$	0.2674

e^{-x}&ln(x)	0.6393	e^{-x}&ln(x)	0.7236	e^{-x}&ln(x)	0.248
e^{-x}&e^{-x}	0.5092	e^{-x}&e^{-x}	0.7100	e^{-x}&e^{-x}	0.2344
e^{-x}&$x^{1/2}$	0.5344	e^{-x}&$x^{-1/2}$	0.7367	e^{-x}&$x^{-1/2}$	0.2611
$x^{1/2}$&ln(x)	0.6404	$x^{1/2}$&ln(x)	0.7488	$x^{1/2}$&ln(x)	0.2491
$x^{1/2}$&e^{-x}	0.5103	$x^{1/2}$&e^{-x}	0.7352	$x^{1/2}$&e^{-x}	0.2355
$x^{1/2}$&$x^{1/2}$	0.5355	$x^{1/2}$&$x^{-1/2}$	0.7619	$x^{1/2}$&$x^{-1/2}$	0.2622

Supplementary Table 4. The coefficient of determination R^2 in 2nd round of least square regressions with three fingerprint and corresponding three best prototypical functions selected from Supplementary Table 2.

Descriptor	R^2	Descriptor	R^2	Descriptor	R^2
ln(x)&ln(x)&ln(x)	0.8768	e^{-x}&ln(x)&ln(x)	0.8706	$x^{1/2}$&ln(x) & ln(x)	0.8716
ln(x)&ln(x) & e^{-x}	0.8632	e^{-x}&ln(x)&e^{-x}	0.8570	$x^{1/2}$&ln(x) & e^{-x}	0.858
ln(x)&ln(x) & $x^{-1/2}$	0.8899	e^{-x} &ln(x)&$x^{-1/2}$	0.8837	$x^{1/2}$&ln(x) & $x^{-1/2}$	0.8847
ln(x)&e^{-x}&ln(x)	0.7467	e^{-x} &e^{-x}&ln(x)	0.7404	$x^{1/2}$&e^{-x}& ln(x)	0.7415
ln(x)&e^{-x}&e^{-x}	0.7331	e^{-x} &e^{-x}&e^{-x}	0.7268	$x^{1/2}$&e^{-x}&e^{-x}	0.7279
ln(x)&e^{-x}&$x^{-1/2}$	0.7598	e^{-x} &e^{-x}&$x^{-1/2}$	0.7535	$x^{1/2}$&e^{-x} & $x^{-1/2}$	0.7546
ln(x)&$x^{1/2}$&ln(x)	0.7719	e^{-x} &$x^{1/2}$&ln(x)	0.7656	$x^{1/2}$&$x^{1/2}$& ln(x)	0.7667
ln(x)&$x^{1/2}$&e^{-x}	0.7583	e^{-x} &$x^{1/2}$&e^{-x}	0.752	$x^{1/2}$&$x^{1/2}$&e^{-x}	0.7531
ln(x)&$x^{1/2}$&$x^{-1/2}$	0.7850	e^{-x} &$x^{1/2}$&$x^{-1/2}$	0.7787	$x^{1/2}$&$x^{1/2}$&$x^{-1/2}$	0.7798

Taking ln(x) function and three fingerprints as the example, some details of LSR are provided in the following. Here, we normalize the three parameters by taking the ratio of the filler and the matrix. So x_1, x_2, and x_3 represent the normalized value of dielectric constant, electrical conductivity, Young's modulus and breakdown strength, as shown below:

$$x_1 = \varepsilon_{\text{filler}} / \varepsilon_{\text{matrix}}, \quad x_2 = \sigma_{\text{filler}} / \sigma_{\text{matrix}}, \quad x_3 = Y_{\text{filler}} / Y_{\text{matrix}}$$

Similarly, y denotes the ratio of the breakdown strength of the nanocomposite and the pure polymer, as written by

$$y = E_{\text{b}}^{\text{composite}} / E_{\text{b}}^{\text{matrix}}$$

Therefore, we use $\ln(x_1)$, $\ln(x_2)$, and $\ln(x_3)$ as fingerprints to make least square regressions. The dependence and residual case order plot are shown in Supplementary Figures 2 and 3

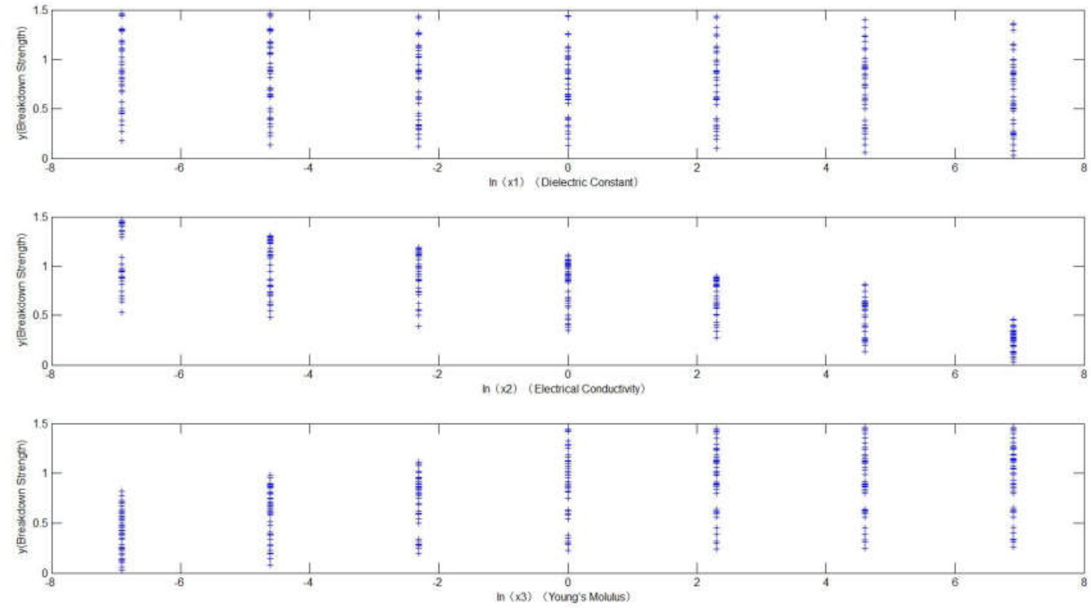

Supplementary Figure 2 Dependences of y on $\ln(x_1)$, $\ln(x_2)$, and $\ln(x_3)$.

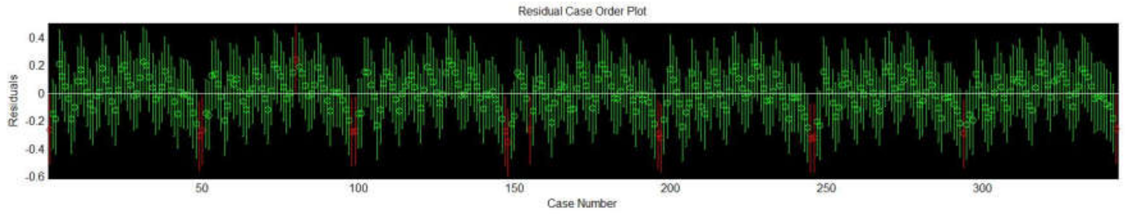

Supplementary Figure 3 The residual case order plot.

Next, the interactions among fingerprints with corresponding best prototypical function are considered based on the results of the 2nd round of regressions, and the results are shown in Supplementary Table 5. When considering all interactions, the regression shows a best R^2=0.9099. The interaction between σ and Y has a close significant dependence with R^2=0.9093 with a coefficient of 0.001 for ln(x) & $x^{-1/2}$ term. Therefore, we choose the latter one for simplicity with the expression given by:

$$\frac{E_b^{\text{composite}}}{E_b^{\text{matrix}}}=0.9058-0.01175\ln\frac{\varepsilon_{\text{filler}}}{\varepsilon_{\text{matrix}}}-0.06767\ln\frac{\sigma_{\text{filler}}}{\sigma_{\text{matrix}}}-0.01640\left(\frac{Y_{\text{filler}}}{Y_{\text{matrix}}}\right)^{-1/2}+0.001\left(\frac{Y_{\text{filler}}}{Y_{\text{matrix}}}\right)^{-1/2}\ln\frac{\sigma_{\text{filler}}}{\sigma_{\text{matrix}}}$$

Then, we use additional phase-field simulations and experimental measurements to verify the machine learning results. The material parameters used in the additional phase-field simulations are listed in Supplementary Table 6. The experimental fabrication process of Al_2O_3/P(VDF-HFP) nanocomposite in this work is shown below.

Supplementary Table 5. The coefficient of determination R^2 in the 3rd round least square regressions with consideration of interactions among descriptors. The total analytical function is

$$y = \beta_0 + \beta_1 \ln(x_1) + \beta_2 \ln(x_2) + \beta_3 (x_3)^{-1/2} + \beta_4 \ln(x_1)\ln(x_2) + \beta_5 \ln(x_1)(x_3)^{-1/2} + \beta_6 \ln(x_2)(x_3)^{-1/2} + \beta_7 \ln(x_1)\ln(x_2)(x_3)^{-1/2}$$

	None	ε & σ	ε & Y	σ & Y	ε & σ &Y	All
R^2	0.8899	0.8899	0.8900	0.9093	0.8900	0.9099
β_0	0.9058	0.9058	0.9058	0.9058	0.9058	0.9058
β_1	-0.01175	-0.01175	-0.01085	-0.01175	-0.01175	-0.01085
β_2	-0.06104	-0.06103	-0.06103	-0.06767	-0.06103	-0.06767
β_3	-0.0164	-0.0164	-0.0164	-0.0164	-0.0164	-0.0164
β_4	/	0.000003	/	/	/	0.00002
β_5	/	/	0.00013	/	/	-0.00014
β_6	/	/	/	0.001	/	-0.00013
β_7	/	/	/	/	0.000016	0.001

Supplementary Table 6. Material parameters of nanofillers[6-10] used in additional phase-field simulations for verifying the machine learning results.

	Dielectric constant /	Electrical conductivity (S m^{-1})	Young's modulus (GPa)	$E_b^{\text{composite}} / E_b^{\text{polymer}}$		
				Phase-field method	Machine learning	Experimental Results
P(VDF-HFP)	13.5	2.12e-10	0.982	1	0.8	/
Al_2O_3/P(VDF-HFP)	10	1.0e-12	300	1.428	1.338	1.15
TiO_2/P(VDF-HFP)	48	1e-10	230	1.01	1.029	/

SiO_2/P(VDF-HFP)	4	1e-13	280	1.40	1.484	/
$BaTiO_3$/P(VDF-HFP)	300	1e-8	70	0.64	0.684	0.686[11]
$SrTiO_3$/P(VDF-HFP)	200	1e-9	70	0.86	0.83	/
MgO/P(VDF-HFP)	9.7	5e-15	100	1.52	1.62	1.58[12]
BCZT/P(VDF-HFP)	400	5e-7	70	0.62	0.583	0.667[13]

Supplementary Discussion

The Local Distributions of Energy Densities

More energy distributions corresponding to the breakdown states in Supplementary Figure 1 are displayed in Supplementary Figures 4, 5 and 6. The distributions of strain induced by the electric force are shown in Supplementary Figure 7.

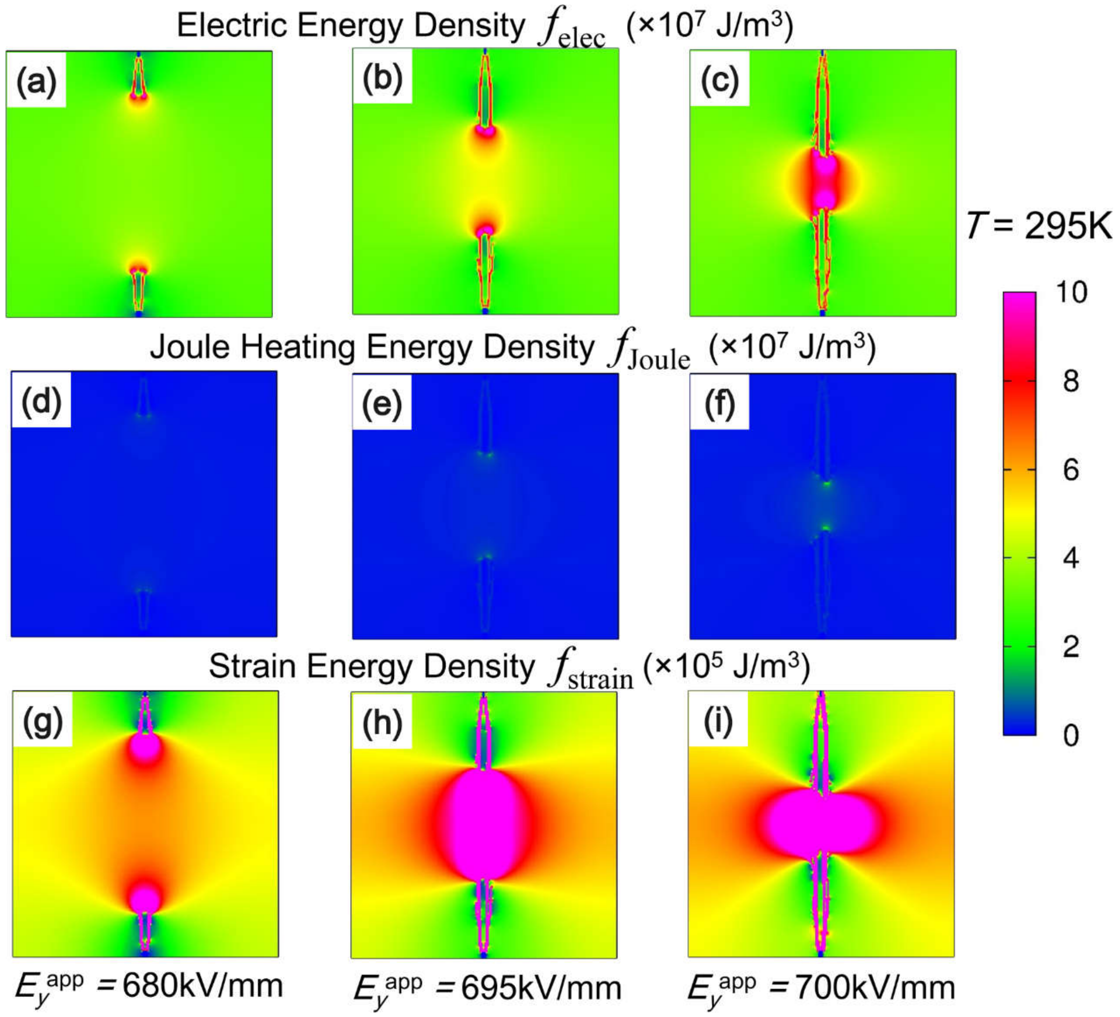

Supplementary Figure 4 Energy density distributions during breakdown at 295K (a), (b), (c) The local distributions of electric energy density, (d), (e), (f) Joule heat energy density, (g), (h), (i) strain energy density (g)-(i) at 295K, corresponding to the states in Figs. 1(a),1(b) and 1(c).

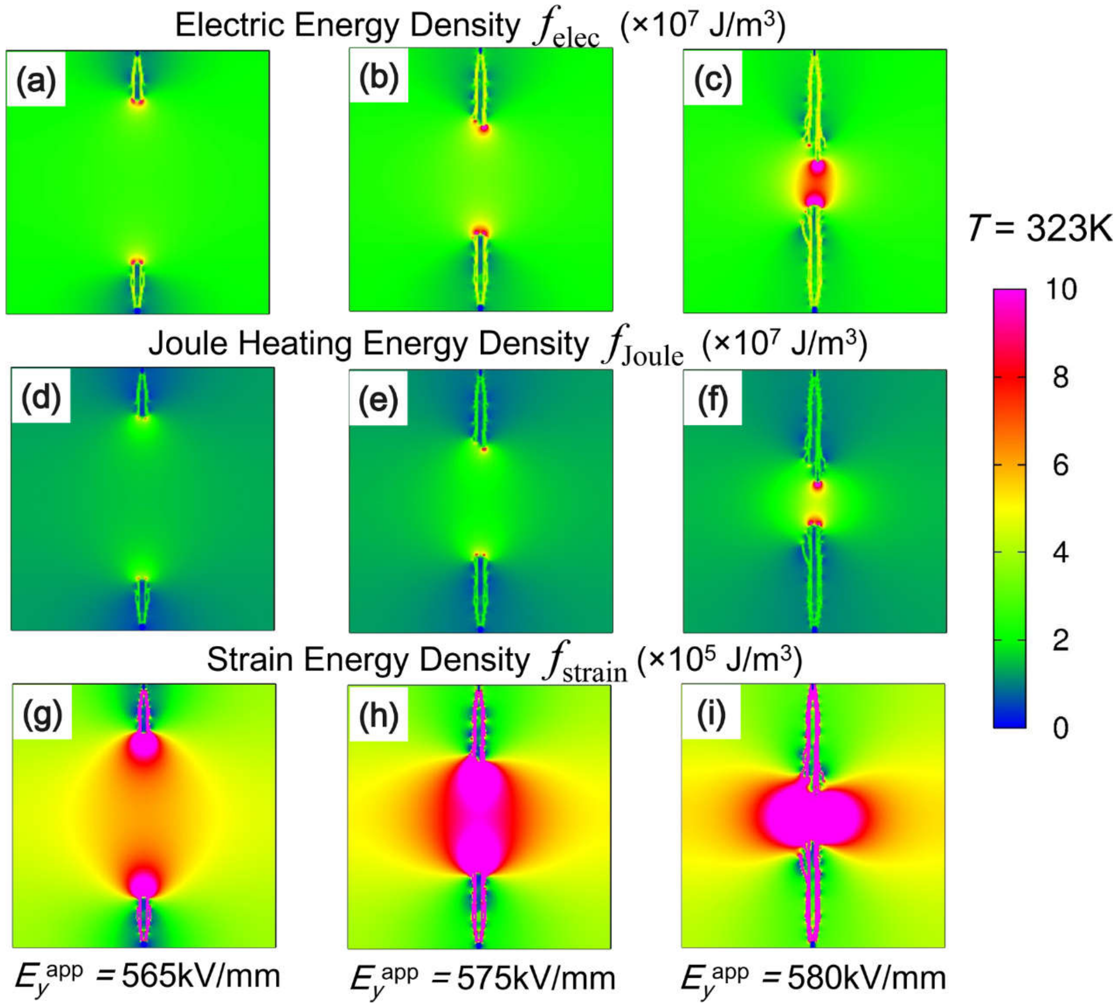

Supplementary Figure 5 Energy density distributions during breakdown at 323K (a), (b), (c) The local distributions of electric energy density, (d), (e), (f) Joule heat energy density, (g), (h), (i) strain energy density (g)-(i) at 323K, corresponding to the states in Figs. 1(d),1(e) and 1(f).

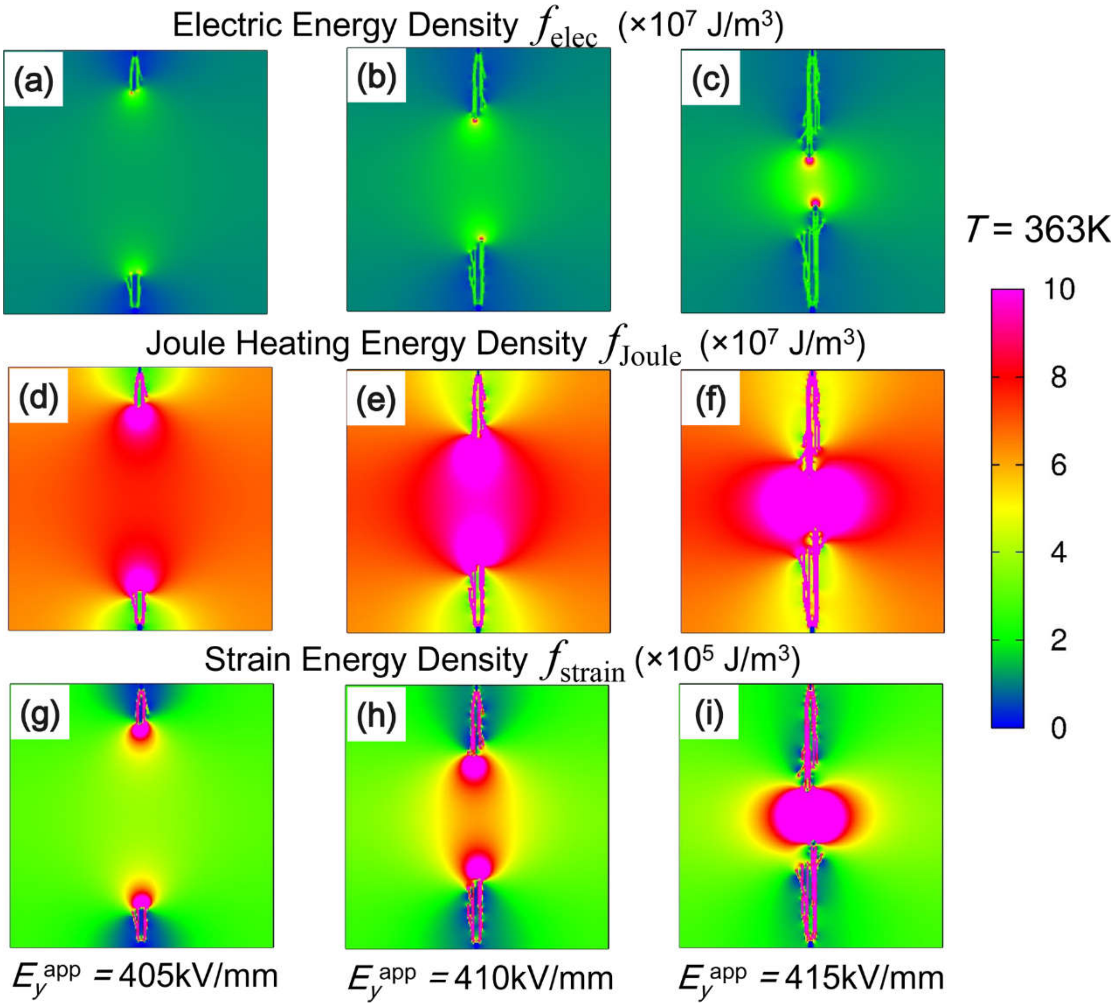

Supplementary Figure 6 Energy density distributions during breakdown at 363K (a), (b), (c) The local distributions of electric energy density, (d), (e), (f) Joule heat energy density, (g), (h), (i) strain energy density (g)-(i) at 363K, corresponding to the states in Figs. 1(g), 1(h) and 1(i).

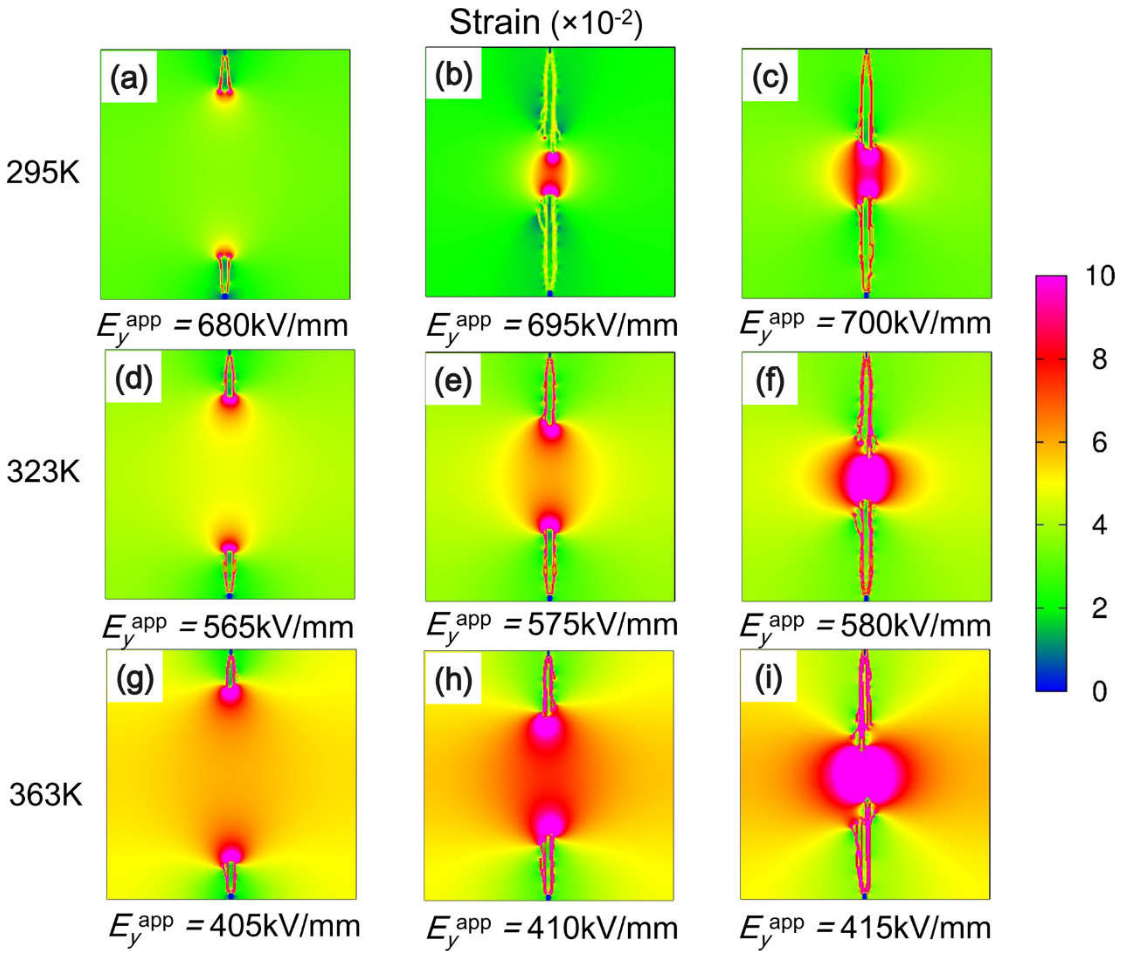

Supplementary Figure 7 Electromechanical strain distributions during breakdown at different temperatures. The local distributions of strain at (a), (b), (c) 295K, at (d), (e), (f) 323K, at (g), (h), (i) 363K, corresponding to the states in Figs. 1(a)-1(i).

Experimental Results of Al_2O_3/P(VDF-HFP) Nanocomposite

In this work, Al_2O_3/P(VDF-HFP) nanocomposites were experimentally fabricated using our previous fabrication method[11,14]. Chemicals were obtained from the following commercial sources and used without further purification: Al_2O_3 (China National Chemicals Corporation Ltd.), poly(vinylidene fluoride-co-hexafluoropropylene) (P(VDF-HFP), Arkema, France, Kynar Flex 2801 with 10 wt %HFP). P(VDF-HFP) powder was thoroughly dissolved in a mixed solvent of N,N-dimethylformamide (DMF) and acetone. With the aid of ultrasonic treatment, Al_2O_3 nanoparticles were then dispersed into the P(VDF-HFP) solution with a volume fraction 5%. Then, electrospinning and hot pressing process were performed. Finally, a ~15μm thick Al_2O_3/P(VDF-HFP) nanocomposite was obtained. More details of fabrication are same to our previous works[11,14]. The SEM image was shown in Supplementary Figure 8(a). The measured breakdown strengths of the prepared P(VDF-HFP) pure polymer and Al_2O_3/P(VDF-HFP) nanocomposites were plotted in a Weibull distribution diagram, which indicates breakdown strengths of 561.2 V mm^{-1} and 647.6 kV mm^{-1} for the pure P(VDF-HFP) polymer and Al_2O_3/P(VDF-HFP) nanocomposites, respectively (Supplementary Figure 8(b)).

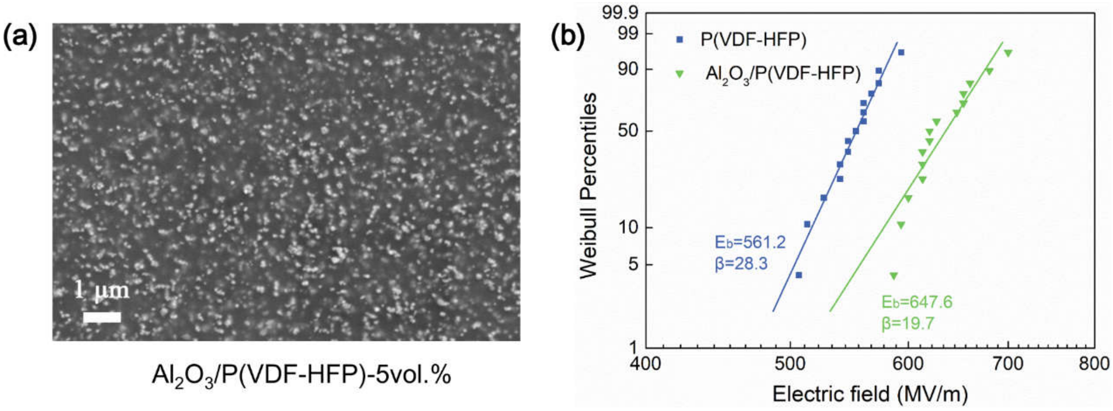

Supplementary Figure 8 Experimental results of Al_2O_3/P(VDF-HFP). (a) SEM image of Al_2O_3/P(VDF-HFP) nanocomposite with 5vol % fillers. (b) Weibull distributions of breakdown strengths of pure P(VDF-HFP) and Al_2O_3/P(VDF-HFP) nanocomposites.

Back-propagation neural network (BPNN) approach

The back-propagation neural network (BPNN) approach is performed based on gradient descent algorithm. The data calculated by phase-field simulations are used as inputs and are divided into training set, validation set and test set with the proportion of 75%, 15% and 15%, respectively. The transfer functions of 10 hidden layers and 1 output layer in this framework are sigmoidal function and linear function, respectively. Supplementary Figures 9 and 10 give the assessment results of the accuracy and reliability of this machine learning method. It can be seen that the training process meet the requirement of error after 82 epochs. Correlation coefficients shown in Supplementary Figure 10 indicate that the prediction results from BPNN machine learning are reliable, with almost all $R>0.99$. We present the comparison results of breakdown strengths calculated by the phase-field model and predicted by BPNN in Supplementary Figure 11, which show a higher coefficient of determination $R^2 = 0.983$ than that of LSR in Fig. 6b.

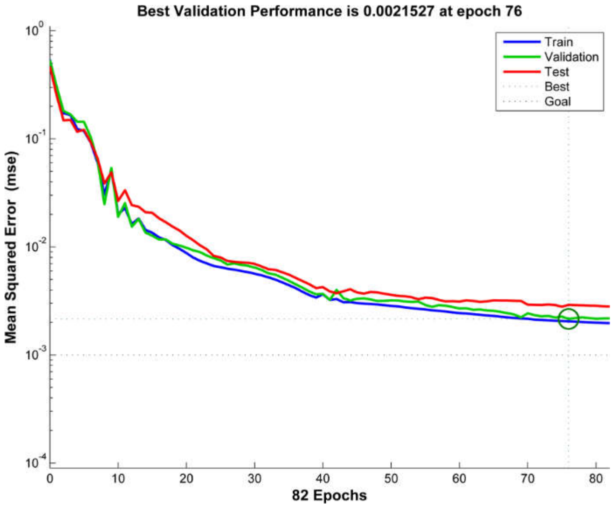

Supplementary Figure 9 The changes of mean squared error (MSE) of different sets.

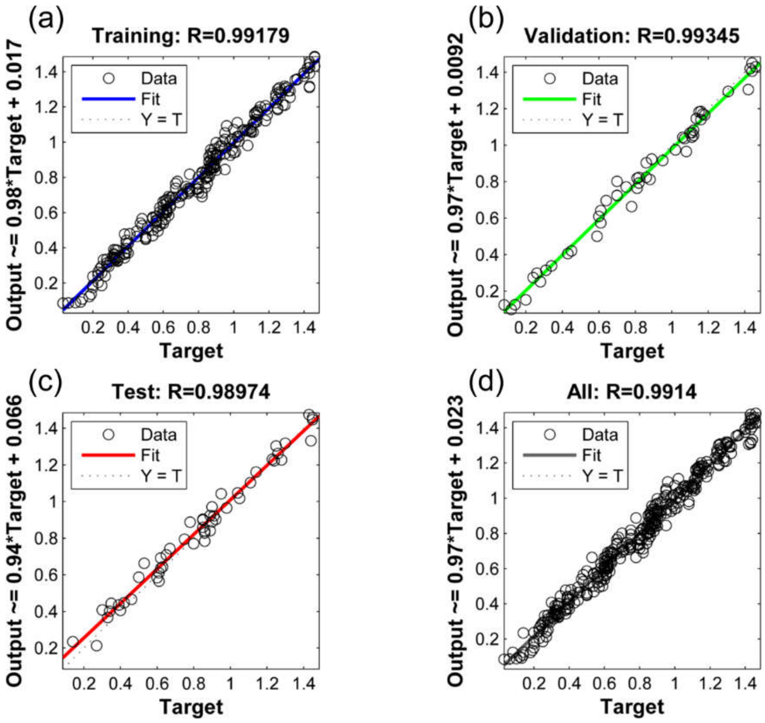

Supplementary Figure 10 Regression plots of different sets of outputs and targets: (a) training set (b) validation set (c) testing set and (d) all data. Here, Target in x axis denotes the original data (phased-field simulation results) and Output in y axis represents the predicted results by BPNN. Correlation coefficient *R* explains the degree of correlation between the target and output.

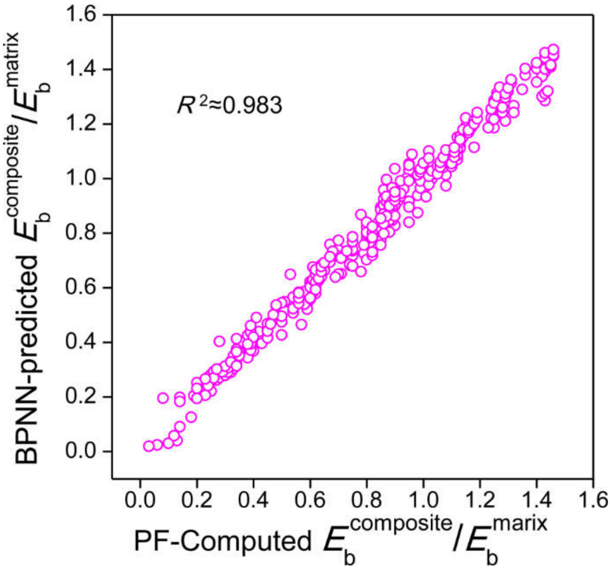

Supplementary Figure 11 Comparisons of breakdown strengths between the phase-field calculated results and the predicted results by back-propagation neural network approach.

Supplementary References

1. Dissado, L. A. & Fothergill, J. C. *Electrical degradation and breakdown in polymers*. IET (1992).

2. Kumar, A., Sharma, R., Suresh, M., Das, M. K. & Kar, K. K. Structural and ion transport properties of lithium triflate/poly (vinylidene fluoride-co-hexafluoropropylene)-based polymer electrolytes: Effect of lithium salt concentration. *J Elastom. Plast.* **49**, 513-526 (2017).

3. Zhou, X. et al. Electrical breakdown and ultrahigh electrical energy density in poly (vinylidene fluoride-hexafluoropropylene) copolymer. *Appl. Phys. Lett.* **94**, 162901 (2009).

4. McPherson, J. W., Kim, J., Shanware, A., Mogul, H. & Rodriguez, J. Trends in the ultimate breakdown strength of high dielectric-constant materials. *IEEE trans. electron Dev.* **50**, 1771-1778 (2003).

5. Biner, S. & Hu, S. Y. Simulation of damage evolution in composites: A phase-field model. *Acta Mater.* **57**, 2088-2097 (2009).

6. Shi, T., Chen, Y. & Guo, X. Defect chemistry of alkaline earth metal (Sr/Ba) titanates. *Prog. Mater. Sci.* **80**, 77-132 (2016).

7. Klein, A. Interface properties of dielectric oxides. *J Am. Ceram. Soc.* **99**, 369-387 (2016).

8. Wang, J.-J. et al. Defect chemistry and resistance degradation in Fe-doped $SrTiO_3$ single crystal. *Acta Mater.* **108**, 229-240 (2016).

9. Fredin, L. A., Li, Z., Ratner, M. A., Lanagan, M. T. & Marks, T. J. Enhanced energy storage and suppressed dielectric loss in oxide core–shell–polyolefin nanocomposites by moderating internal surface area and increasing shell thickness. *Adv. Mater.* **24**, 5946-5953 (2012).

10. Nelson, J. K. *Dielectric polymer nanocomposites*. Springer (2010).

11. Jiang, J. et al. Tuning Phase Composition of Polymer Nanocomposites toward High Energy Density and High Discharge Efficiency by Nonequilibrium Processing. *ACS Appl. Mater. Interfaces* **9**, 29717-29731 (2017).

12. Chen, S.-s. et al. Enhanced breakdown strength and energy density in PVDF nanocomposites with functionalized MgO nanoparticles. *RSC Adv.* **6**, 33599-33605 (2016).

13. Luo, B., Wang, X., Wang, Y. & Li, L. Fabrication, characterization, properties and theoretical analysis of ceramic/PVDF composite flexible films with high dielectric constant and low dielectric loss. *J Mater. Chem. A* **2**, 510-519 (2014).

14. Zhang, X. et al. Polymer Nanocomposites with Ultrahigh Energy Density and High Discharge Efficiency by Modulating their Nanostructures in Three Dimensions. *Adv. Mater.* **30**, 1707269 (2018).
